# Identification of functional single nucleotide polymorphisms in the branchpoint site

**DOI:** 10.1186/s40246-017-0122-6

**Published:** 2017-11-09

**Authors:** Hung-Lun Chiang, Jer-Yuarn Wu, Yuan-Tsong Chen

**Affiliations:** 1 Institute of Clinical Medicine, National Yang-Ming University, Taipei, Taiwan; 2 Institute of Biomedical Sciences, Academia Sinica, Taipei, Taiwan; 3 Graduate Institute of Chinese Medical Science, China Medical University, Taichung, Taiwan; 4 Department of Pediatrics, Duke University Medical Center, Durham, USA

**Keywords:** RNA splicing, Single nucleotide polymorphism, Branchpoint site, Minigene

## Abstract

**Background:**

The human genome contains millions of single nucleotide polymorphisms (SNPs); many of these SNPs are intronic and have unknown functional significance. SNPs occurring within intron branchpoint sites, especially at the adenine (A), would presumably affect splicing; however, this has not been systematically studied. We employed a splicing prediction tool to identify human intron branchpoint sites and screened dbSNP for identifying SNPs located in the predicted sites to generate a genome-wide branchpoint site SNP database.

**Results:**

We identified 600 SNPs located within branchpoint sites; among which, 216 showed a change in A. After scoring the SNPs by counting the As in the ± 10 nucleotide region, only four SNPs were identified without additional As (rs13296170, rs12769205, rs75434223, and rs67785924). Using minigene constructs, we examined the effects of these SNPs on splicing. The three SNPs (rs13296170, rs12769205, and rs75434223) with nucleotide substitution at the A position resulted in abnormal splicing (exon skipping and/or intron inclusion). However, rs67785924, a 5-bp deletion that abolished the branchpoint A nucleotide, exhibited normal RNA splicing pattern, presumably using two of the downstream As as alternative branchpoints. The influence of additional As on splicing was further confirmed by studying rs2733532, which contains three additional As in the ± 10 nucleotide region.

**Conclusions:**

We generated a high-confidence genome-wide branchpoint site SNP database, experimentally verified the importance of A in the branchpoint, and suggested that other nearby As can protect branchpoint A substitution from abnormal splicing.

**Electronic supplementary material:**

The online version of this article (10.1186/s40246-017-0122-6) contains supplementary material, which is available to authorized users.

## Background

Precursor messenger RNA (pre-mRNA) splicing is essential for gene expression in eukaryotes [1–3]. Splicing comprises a two-step trans-esterification reaction of intron removal and exon ligation. Splicing depends on the spliceosome, which is a large complex of small nuclear ribonucleoproteins (snRNPs; U1, U2, U4/U6, and U5) and non-snRNPs; these components recognize the target sequence and assemble on the pre-mRNA [4]. The intronic target sequences include a 5′ donor site, a 3′ acceptor site, a polypyrimidine tract (PPT) upstream of the 3′ acceptor, and a branchpoint site upstream of the PPT. The branchpoint contains a conserved splicing signal important for spliceosome assembly and lariat intron formation, with a consensus sequence (YNCTRAY, which differs slightly between species; Y is pyrimidine, N is any nucleotide, and R is purine) [5]. Tools to predict branchpoint sites based on the consensus sequence have been developed [6–10]; more recently, an NGS-based genome-wide study of splicing branchpoints was published [11–13].

Within the consensus branchpoint site sequence YNCTRAY, the well conserved A appears to be the most important one. A previous report showed that IVS4,-22A>G in the *LCAT* gene, which is an A to G change at the splicing branchpoint, resulted in intron inclusion and exon skipping of the mRNA and caused the Fish-eye disease [14]. There is also a report suggesting that mutations in the branchpoint sequence, especially the adenine (A) may result in aberrant pre-mRNA splicing and give rise to human genetic disorders [15].

There are millions of SNPs in the human genome; many are intronic, and have unknown functional significance. SNPs at the intron branchpoint sites, especially the adenine (A) nucleotide, would presumably affect splicing; however, this has not been systematically studied. It is therefore desirable to create a genome-wide branchpoint site SNP database, and perform functional analysis.

In the present study, we used an in silico splicing prediction program for branchpoint site prediction and combined its predictions with dbSNP data, to create a genome-wide branchpoint site SNP dataset. We experimentally verified the importance of A in the branchpoint, and further suggested that other nearby As may also influence RNA splicing.

## Methods

### Creating a dataset of SNPs located within branchpoint sites

All exon (*n* = 404,454) and intron (*n* = 363,190) sequences of the human genes were collected (human 1000genome v37), and the SROOGLE tool, which is based on two different algorithms, was used to predict branchpoint sites [8]. We were able to predict 338,787 (93.3%) branchpoint sites as output. Next, we screened NCBI’s dbSNP for candidate SNPs located within the set of predicted branchpoint sites. Because adenine is the most important nucleotide at the branchpoint site, and 90% of branchpoint sites are upstream 19–37 bp from the 3′ acceptor [12, 13], we scored each SNP by the number of adenines found in the ± 10 nucleotide region (20 nucleotides total) surrounding the SNP. The SNPs identified in the predicted branchpoint sites and reported lariat sequences associated with these SNPs [12] are tabulated in Additional file 1.

### Cell lines and genotyping

293T cells were obtained from The Bioresource Collection and Research Center (Hsinchu, Taiwan). Randomly selected EBV-transformed normal control B cell lines (*n* = 96) were obtained from the Taiwan Han Chinese Cell and Genome Bank [16]. Genomic DNA was extracted from the cell lines using the Gentra Puregene® Blood Kit (Gentra Systems, MN, USA) and genotyped for the SNPs of interest (*XPC* rs2733532, *PIP5KL1* rs13296170, *CYP2C19* rs12769205, *MYH11* rs75434223, and *KLC3* rs67785924), to identify cell lines carrying different branchpoint site SNP alleles (Table 1). The primer sequences are provided in Additional file 2.

**Table 1:** Selected splice-site SNPs for functional studies

Chromosome	Position	Position	Gene name	SNP ID	Alleles	SNP ± 10 nucleotides sequence	Allele frequency
3	14187698	14187699	*XPC*	rs2733532	A/G	TCTGATTACT*A*ACCCTCGCCT	A = 0.363 G = 0.637
9	130689507	130689508	*PIP5KL1*	rs13296170	A/C	GGCCTCCCTC*A*CTCCCTGTCC	A = 1 C = 0
10	96535124	96535125	*CYP2C19*	rs12769205	A/G	TCTCCCTCCT*A*GTTTCGTTTC	A = 0.670 G = 0.330
16	15835555	15835556	*MYH11*	rs75434223	A/C	CGTGGGGCTC*A*CCCGCCTCCT	A = 1 C = 0
19	45854507	45854508	*KLC3*	rs67785924	−/ACCTC	CTTGCCCCTC*A*CCTCCCCTCC	− = 0.079 ACCTC = 0.921

### Minigene constructs

**Fig. 1:**
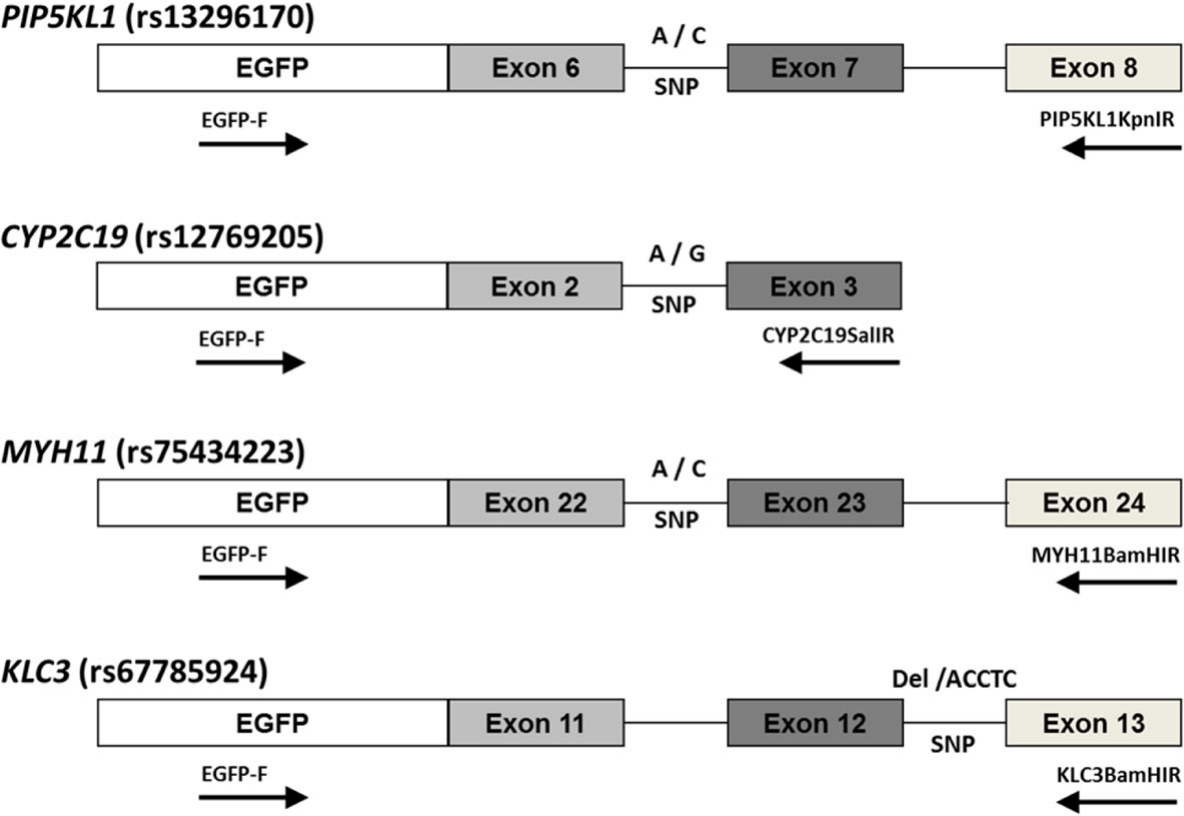
Schematic representation of minigene constructs. SNPs in the branching point sites are indicated. Locations and orientations of RT-PCR primers (arrows) are shown. See Additional file 2: Table S2 for primer sequences

Minigene constructs (Fig. 1) encompassing exons/introns of interest were prepared by amplifying introns and exons from genomic DNA; the amplified regions comprised *PIP5KL1* (chr9:130688147-130689612; 1466 bp), *CYP2C19* (chr10:96534815-96535296; 482 bp), *MYH11* (chr16:15833924-15835748; 1825 bp), and *KLC3* (chr19:45853898-45854704; 807 bp). The amplified minigenes were cloned into pJET1.2/blunt cloning vector (Thermo Scientific, Waltham, MA, USA), and subsequently sub-cloned into pEGFP-C1 vector (Additional file 2 indicates each restriction enzyme site). *PIP5KL1* and *MYH11’*s SNPs and *KLC3*’s seventh and eighth adenine substitution were used the GeneArt™ Site-Directed Mutagenesis System (Thermo Scientific, Waltham, MA, USA) with mutagenesis primers (Additional file 2). The complete sequences of the minigene constructs were confirmed by Sanger sequencing. Transient transfections of minigene constructs in 293T cells were performed using TransIT®-2020 transfection reagent (Mirus Bio, Inc., Madison, WI, USA). To isolate total RNA, the cells were harvested in TRIzol® reagent 24 h later, following the manufacturer’s instructions to isolate total RNA.

### Reverse transcription-PCR (RT-PCR)

Each cDNA was prepared from 2 μg total RNA, which was extracted from different minigenes of transfected cells and EVB-transformed B cells, using SuperScript®III reverse transcriptase, with oligo(dT)12–18 as primer, following the manufacturer’s protocol (Invitrogen, CA, USA). All RT-PCR products were gel extracted and sequenced to confirm normal splicing, intron inclusion, and exon skipping forms.

## Results

We identified 600 SNPs at the branchpoint sites; among these SNPs, 216 showed a change in adenine. After scoring the SNPs by counting the As in the ± 10 nucleotide region, only four SNPs were identified without any additional As; 17 SNPs had one additional A, and 29 SNPs had two additional As (Additional file 1).

The four SNPs identified without any additional As in the ± 10 nucleotide region were rs13296170, rs12769205, rs75434223, and rs67785924; these SNPs were the candidates most likely to affect RNA splicing (Table 1). rs13296170, rs12769205, rs75434223, and rs67785924 are located on *PIP5KL1* intron6, *CYP2C19* intron2, *MYH11* intron22, and *KLC3* intron12, respectively (Fig. 1). These SNPs were further investigated for their functional significance. Minigenes containing the SNPs of interest were built using 3 exons and 2 introns, except for the *CYP2C19* SNP, for which two exons and one intron were used, because intron 3 of *CYP2C19* is large in size (4.9 kb).

**Fig. 2:**
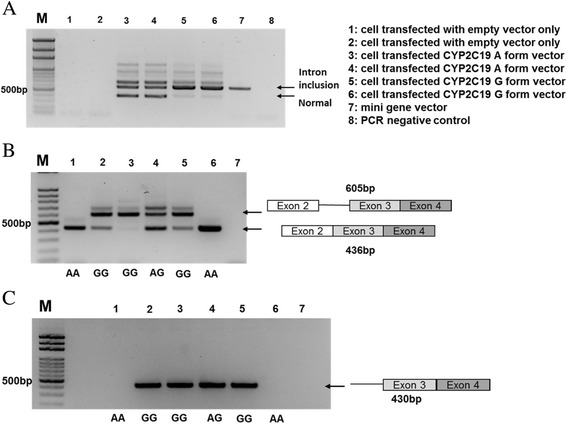
*CYP2C19* alternative splicing forms in minigene-transfected 293T and in EBV-transformed B cells carrying different genotypes at SNP rs12769205. *CYP2C19* RT-PCR was performed with **a** 293T cells transfected with minigene of rs12769205, genotype A or G, using EGFP-F and *CYP2C19 * SalIR primers, and **b** cDNA from B cells carrying different genotypes AA, AG, and GG at rs12769205 position using *CYP2C19 * ex2F and *CYP2C19 * ex4R primers or **c** cDNA from B cells using *CYP2C19 * in2F and *CYP2C19 * ex4R as primer set. Marker represents the 100-bp DNA ladder and indicated 500-bp site. See Additional file 2: Table S2 for primer sequences

RT-PCR of cDNAs prepared from 293T cells transfected with different minigene constructs showed that the rs12769205 A allele produced three bands (normal spliced, intron inclusion, and hybrid forms) when A was substituted with guanine (G) in *CYP2C19*, which spliced majorly in the intron inclusion form with lesser normal form (Fig. 2a). Since this construct comprised two exons and one intron, to make sure there was no exon skipping, we examined mRNA from EBV-transformed B cells carrying different genotypes for the spliced forms. The results showed that B cells had genotype AA spliced in the normal form, AG spliced equally in the normal and intron inclusion forms, and GG spliced mostly in the intron inclusion form (Fig. 2b). The results were further confirmed by using another set of primers such that the forward primer was located on intron 2, and it was noted that AG and GG genotypes spliced in the intron inclusion form (Fig. 2c).

**Fig. 3:**
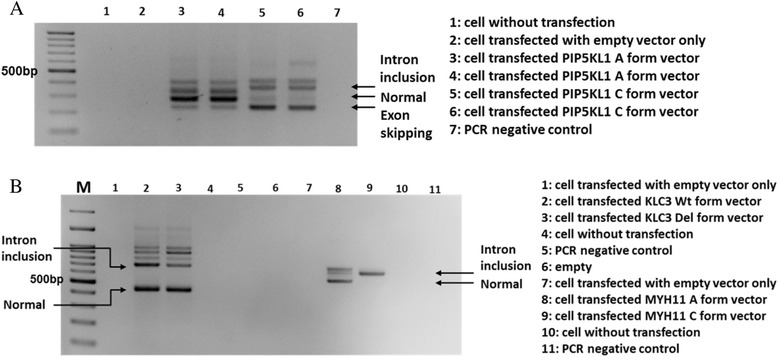
*PIP5KL1*, *KLC3*, and *MYH11* alternative splicing in minigene-transected 293T cell. RT-PCR was performed in minigene-transfected 293T cells. **a** *PIP5KL1*; rs13296170, genotype A or C, was using EGFP-F and *PIP5KL1* KpnIR primers. **b** *KLC3* (land 1~5); rs67785924, genotype ACCTC (Wt) or deletion (Del), with EGFP-F and *KLC3* BamHIR primers, and *MYH11* (land 7~11); rs75434223, genotype A or C, was using EGFP-F and *MYH11* BamHIR primers. Marker represents the 100-bp DNA ladder and indicated 500-bp site. See Additional file 2: Table S2 for primer sequences

We also studied the minigene constructs for the other three SNPs. When A was substituted with cytosine (C) in rs13296170, *PIP5KL1* spliced mostly into the exon skipping form and somewhat into the intron inclusion form, but not into the normal-spliced RNA form (Fig. 3a, lanes 5 and 6). While rs75434223 substituted A with C, *MYH11* spliced into the intron inclusion form, and not into the normal spliced form (Fig. 3b, lanes 8 and 9).

The SNP rs67785924 in *KLC3* has a normal (wild type) allele containing A and a deletion allele with five missing nucleotides, ACCTC. Both alleles produced normal spliced form, and some intron inclusion form. The level of intron inclusion form in the deletion allele was actually less than that in the normal A allele (Fig. 3b, lanes 2 and 3).

**Fig. 4:**
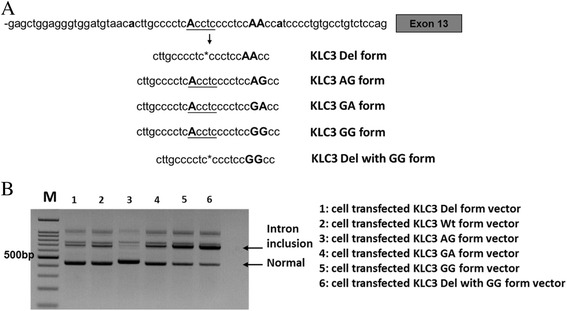
Nucleotide sequences of *KLC3* intron 12 and alternative splicing forms in minigene-transected 293T cell. **a** Nucleotide sequences at 3′part of the *KLC3* intron 12. Possible splicing branchpoint adenine is indicated by bold and uppercase, SNP rs67785924 is underlined, and asterisk shown after deletion sequence. The following shown each A substituted minigene. **b** RT-PCR was performed in 293T cells transfected with different *KLC3* adenine substitution minigene constructs: ACCTC (Wt); ACCTC deletion (Del); Wt with AG (AG form); Wt with GA (GA form); Wt with GG (GG form) and Del with GG form. Marker represents the 100-bp DNA ladder and indicated 500-bp site. See Additional file 2: Table S2 for primer sequences

To understand why the deletion allele that did not contain branchpoint A still produces the normally spliced form, we checked the nearby intron sequence and found two other As located at the seventh and eighth nucleotides from the branchpoint A (Fig. 4a). We performed the branchpoint site prediction analysis using SROOGLE and Human Splicing Finder [9]; both tools predicted that these two nearby As also lie within the potential consensus branchpoint site sequence and can be used as alternative branchpoints in the deletion allele. We then tested the influence of these two nearby As on splicing using minigene constructs (Fig.4). In the wild-type allele, when the two nearby AA were changed to AG or GA, RNA spliced majorly in the normal form; when changed to GG, there was a decrease in the normal form and an increase in the intron inclusion form. In the deletion allele, when both AA were changed to GG, there was a further decrease in the normal form accompanied with a further increase in the intron inclusion form (Fig.4b). These results suggested other As nearby may serve as alternative branchpoints.

**Fig. 5:**
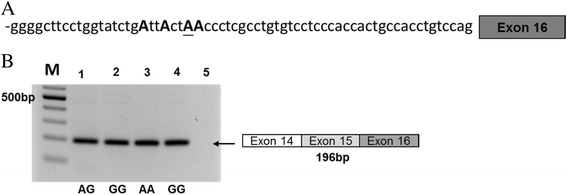
*XPC* alternative splicing in different genotypes of EBV-transformed B cells. **a** Graphic representation 3′part of the *XPC* intron 15 sequences, possible splicing branchpoint adenine is indicated by bold and uppercase, underlining is rs2733532. **b** cDNA from normal control B cells of different genotypes AA, AG, and GG at rs2733532 using *XPC * ex14F and *XPC * ex16R primers. Marker represents the 100-bp DNA ladder and indicated 500-bp site. See Additional file 2: Table S2 for primer sequences

The influence of additional As on splicing was further examined for the branchpoint site SNP rs2733532 A/G, which contains three additional As in the ±10 nucleotide (Fig. 5a). This SNP is located in *XPC* and is reportedly associated with susceptibility to air pollution and childhood bronchitis [17]. In this case, EBV-transformed B cell lines from subjects carrying different genotypes at the branchpoint site, regardless of genotype (AA, AG, or GG), showed only the normally spliced form (Fig. 5b), suggesting that other As can serve as a branchpoint site.

## Discussion

In the present study, we used SROOGLE to predict splice branchpoints and screened dbSNP for SNPs located within the branchpoint sites. Using minigene constructs and, when available, EBV-transformed cell lines carrying different SNP alleles, we experimentally verified that SNPs comprising a change to branchpoint A resulted in abnormal splicing, suggesting that the predicted sites are indeed involved in pre-mRNA splicing, and further confirming the functional importance of A. However, only 20% of the branchpoint sites that we identified had a reported corresponding lariat sequence [12](see Additional file 1). This observation may be understandable given that the number of reported lariat sequences based on next generation sequencing represents only 28% of all introns in the genome [12].

We found only three branchpoint site SNPs that have a single A at the branchpoint site, without additional As nearby. It is possible that organisms evolved to have additional As in the branchpoint site to ensure proper splicing. Additional As in the ± 10 nucleotide region may protect SNPs at the branchpoint A from abnormal splicing, by serving as alternative branchpoints. This mechanism has been demonstrated in the present study for SNP rs67785924 and SNP rs2733532 (Figs. 4 and 5). The latter SNP is located on *XPC* on chromosome 3, and has been reported to be associated with diseases related to air pollution and childhood bronchitis [17]. The risk allele G of the branchpoint SNP A/G resides in the population at a frequency of ~ 0.637. Both the lariat sequence database and our prediction algorithms classified it as a branchpoint site. However, our experiments demonstrated that this branchpoint A to G SNP did not influence splicing; this observation presumably results from the presence of additional nearby As, which serve as alternative splicing sites; this explanation implies that other mechanisms may be involved in this disease association.

Several algorithms and tools have been used to predict branchpoints [6–10], and surprisingly, no branchpoint site SNP database has been reported. Because minigene constructs are time-consuming and not all SNPs in the branchpoint sites have cell lines available for study, in the present study we have tested only five SNPs, and verified their significance. More functional studies are needed to examine the functional significance of other SNPs, especially those SNPs that do not involve A changes at branchpoints.

In conclusion, we have generated a high-confidence genome-wide branchpoint site SNP database, experimentally verified the importance of A in the branchpoint, and suggested that other nearby As may serve as alternative branchpoints and ensure proper pre-mRNA splicing. These results improve upon the prediction of functional SNPs at branchpoint sites, and inform the study of the SNPs at intron branchpoint sites.

## Additional files

Additional file 1: Table S1. Human genome (hg19) coordinates of SNP at branchpoint site. (XLSX 66 kb)

Additional file 2: Table S2. List of primer sequences utilized in the study. (XLSX 10 kb)

## Abbreviations

A: Adenine
C: Cytosine
G: Guanine
PPT: Polypyrimidine tract
pre-mRNA: Precursor messenger RNA
SNP: Single nucleotide polymorphism
snRNPs: Small nuclear ribonucleoproteins
